# Complete mitochondrial genome of the Japanese bumblebee, *Bombus hypocrita hypocrita* (Insecta: Hymenoptera: Apidae)

**DOI:** 10.1080/23802359.2016.1275849

**Published:** 2017-01-11

**Authors:** Mana Nishimoto, Hisashi Okuyama, Takuya Kiyoshi, Tetsuro Nomura, Jun-ichi Takahashi

**Affiliations:** a Department of Life sciences, Kyoto Sangyo University, Kyoto, Japan;; b Department of Zoology, National Museum of Nature and Science, Tokyo, Japan

**Keywords:** Illumina’s Next Seq 500, bumblebee, genetic diversity, conservation

## Abstract

In the present report, we describe the complete mitochondrial genome of the common bumblebee, *Bombus hypocrita hypocrita*, from the Otome Plateau, in Yamanashi Prefecture, Japan. The mitochondrial genome of *B. h. hypocrita* is a circular molecule of 15,795 bp. It contains 13 protein-coding, 22 tRNA and two rDNA genes. The protein-coding genes had ATA, ATG or ATT as the initiation codon and were terminated by the typical stop codon TAA, except for *ND4* and *Cytb*. All the tRNA genes typically formed a cloverleaf secondary structure, except for *trnE and trnS1.*

The Asian orange-tailed bumblebee, *Bombus hypocrita*, is an important bumblebee species for ecosystem and agriculture and is distributed in Far East Asia (Matsumura et al. 2004). Mitochondrial DNA information is very important for species identification and phylogenetic analysis of the bumblebees (Cameron et al. 2007). To our knowledge, this study is the first to successfully determine the sequence of mitochondrial DNA of *B. hypocrita hypocrita* (accession number AP017662).

Adult *B. h. hypocrita* females were collected from the Otome Plateau in Yamanashi Prefecture, Japan (Specimen is stored in the National Museum of Nature and Science, Japan accession number: NSMT-I-HYM74236). The genomic DNA isolated from the worker was sequenced using Illumina’s Next Seq 500 (Illumina). The resultant reads were assembled and analyzed using MITOS web server (Bernt et al. 2013, Germany) and MEGA6 software (Tamura et al. 2013, Japan). Phylogenetic analysis was performed based on the nucleotide sequences of the 13 protein-coding genes using TREEFINDER software (Jobb et al. 2004, Germany).

The *B. h. hypocrita* mitochondrial genome forms a 15,795 bp closed loop. This mitochondrial genome represents a typical hymenopteran mitochondrial genome and matches the *B. h. sapporensis* (Hong et al. 2008; Takahashi et al. 2016) genomes in that it comprises 13 protein-coding, 22 putative tRNA, and two rDNA genes. The average AT content of the *B. h. hypocrita* mitochondrial genome was 85%. Similar to the other bumblebee mitochondrial genomes (Cha et al. 2007; Du et al. 2015), the heavy strand (H-strand) was predicted to contain nine protein-coding and 13 tRNA genes, and the light strand (L-strand) was predicted to contain four protein-coding, nine tRNA and two rDNA genes. The genes, *ATP8* and *ATP6*, shared 19 nucleotides, *ND4* and *ND4L* shared one nucleotide, and *ND6* and *Cytb* shared 13 nucleotide. Six protein-coding genes of the *B. h. hypocrita* mitochondrial genome started with ATA, *ATP6*, *COIII*, *ND4* and *Cytb* started with ATG, and *COII*, *ND5* and *ND4L* started with ATT; these starting codons have been found to be common in the *B. hypocrita* mitochondrial genome (Hong et al. 2008; Takahashi et al. 2016). The stop codon in each of these protein-coding genes was either TAA or TAT, except for *Cytb,* which had TAG, as in other bumblebees.

The complete mitochondrial DNA sequences of 13 mitochondrial protein-coding genes from 14 closely related taxa were analyzed using maximum likelihood method to investigate the phylogenetic relationships within Apinae (Figure 1). The phylogenetic analyses strongly supported the basic topology recoverable from molecular and morphological analyses, grouping the eusocial bee into three major clusters: bumblebee, honeybee and stingless bee. The nucleotide substitution rate between the subspecies of the mitochondrial genomes of *B. h. hypocrita* and *B. h. sapporensis* was 96.1% (14073/14646). In Japan, the population of *B. hypocrita* has reduced due to the genetic pollution of crossing, alien diseases, and competition for nesting-site or feed with the invasive species, *Bombus terrestris* (Goka 2010). The data generated in the present study would help in understanding the genetic diversity of the population and would aid in the conservation of this species.

**Figure 1.**
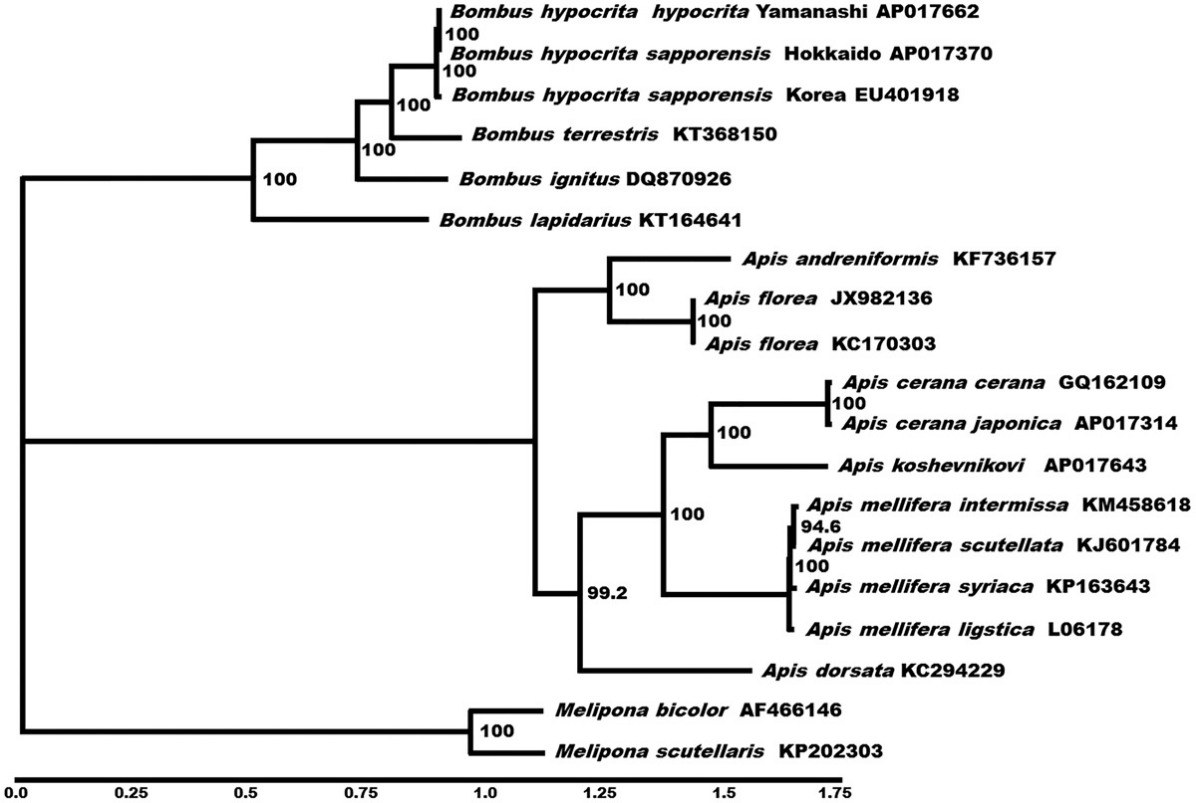
Phylogenetic relationships (determined using the method of maximum likelihood) among the members of Apinae (Order: Hymenoptera) based on the nucleotide sequence of 13 protein-coding genes regions in the mitochondrial genome. The numbers beside the nodes are percentages of 1000 bootstrap values. The *Melipona* species was used as an outgroup. Alphanumeric terms indicate the GenBank accession numbers.

## References

[CIT0001] Bernt M, Donath A, Jühling F, Externbrink F, Florentz C, Fritzsch G, Pütz J, Middendorf M, Stadler PF. 2013 MITOS: Improved de novo Metazoan Mitochondrial Genome Annotation. Mol Phylogenet Evol. 69:313–319. 22982435 10.1016/j.ympev.2012.08.023

[CIT0002] Cameron SA, Hines HM, Williams PH. 2007 A comprehensive phylogeny of the bumble bees (*Bombus*). Biol J Linn Soc. 91:161–188.

[CIT0003] Cha SY, Yoon HJ, Lee EM, Yoon MH, Hwang JS, Jin BR, Han YS, Kim I. 2007 The complete nucleotide sequence and gene organization of the mitochondrial genome of the bumblebee, *Bombus ignitus* (Hymenoptera: Apidae). Gene. 392:206–220. 17321076 10.1016/j.gene.2006.12.031

[CIT0004] Du Q, Bi G, Zhao E, Yang J, Zhang Z, Liu G. 2015 Complete mitochondrial genome of *Bombus terrestris* (Hymenoptera: Apidae). Mitochondrial DNA. Oct5:1–2. 10.3109/19401736.2015.1089568 26437124

[CIT0005] Goka K. 2010 In introduction to the special feature for ecological risk assessment of introduced bumblebees: status of the European bumblebee, *Bombus terrestris*, in Japan as a beneficial pollinator and an invasive alien species. Appl Entomol Zool. 45:1–6.

[CIT0006] Hong MY, Cha SY, Kim DY, Yoon HJ, Kim SR, Hwang JS, Kim KG, Han YS, Kim I. 2008 Presence of several tRNA-like sequences in the mitochondrial genome the bumblebee, *Bombus hypocrita sapporoensis* (Hymenoptera: Apidae). Genes Genom. 30:307–318.

[CIT0007] Jobb G, von Haeseler A, Strimmer K. 2004 TREEFINDER: a powerful graphical analysis environment for molecular phylogenetics. BMC Evol Biol. 4:18. 15222900 10.1186/1471-2148-4-18 PMC459214

[CIT0008] Matsumura C, Yokoyama J, Washitani I. 2004 Invasion status and potential ecological impacts of an invasive alien bumblebee, *Bombus terrestris* L. (Hymenoptera: Apidae) naturalized in Southern Hokkaido, Japan. Global Environmental Research-English Edition. 8:51–66.

[CIT0009] Takahashi J, Nishimoto M, Wakamiya T, Takahashi M, Kiyoshi T, Tsuchida K, Nomura T. 2016 Complete mitochondrial genome of the Japanese bumblebee, *Bombus hypocrita sapporensis* (Insecta: Hymenoptera: Apidae). Mitochondrial DNA Part B. 1:224–225. 10.1080/23802359.2016.1155423 PMC7800672 33473459

[CIT0010] Tamura K, Stecher G, Peterson D, Filipski A, Kumar S. 2013 MEGA6: Molecular evolutionary genetics analysis version 6.0. Mol Biol Evol. 30:2725–2729. 24132122 10.1093/molbev/mst197 PMC3840312

